# Combining Two Technologies for Full Genome Sequencing of Human

**Published:** 2009-10

**Authors:** K.G. Skryabin, E.B. Prokhortchouk, A.M. Mazur, E.S. Boulygina, S.V. Tsygankova, A.V. Nedoluzhko, S.M. Rastorguev, V.B. Matveev, N.N. Chekanov, Goranskaya D.A., A.B. Teslyuk, N.M. Gruzdeva, V.E. Velikhov, D.G. Zaridze, M.V. Kovalchuk

**Affiliations:** 1 Russian Research Centre Kurchatov Institute;; 2 Institute of Carcinogenesis, Blokhin Cancer Research Center, Russian Academy of Medical Sciences;; 3 Bioengineering Center, Russian Academy of Sciences

## Abstract

At present, the new technologies of DNA sequencing are rapidly developing allowing quick and efficient characterisation of organisms at the level of the genome structure. In this study, the whole genome sequencing of a human (Russian man) was performed using two technologies currently present on the market - Sequencing by Oligonucleotide Ligation and Detection (SOLiD™) (Applied Biosystems) and sequencing technologies of molecular clusters using fluorescently labeled precursors (Illumina). The total number of generated data resulted in 108.3 billion base pairs (60.2 billion from Illumina technology and 48.1 billion from SOLiD technology). Statistics performed on reads generated by GAII and SOLiD showed that they covered 75% and 96% of the genome respectively. Short polymorphic regions were detected with comparable accuracy however, the absolute amount of them revealed by SOLiD was several times less than by GAII. Optimal algorithm for using the latest methods of sequencing was established for the analysis of individual human genomes. The study is the first Russian effort towards whole human genome sequencing.

## INTRODUCTION

Progress in the development of novel DNA sequencing technologies allowing rapid and accurate determination of the distinctive features of an individual at the level of the structure of his genome has made genomics one of the most rapidly developing scientific disciplines. At present, three advanced technologies of DNA sequencing are in use: pyrosequencing based on the "sequencing-by-synthesis" principle and commercialized in a next-generation Roche GS-FLX capillary genome sequencing system (454 Life Science Inc./Roche), cyclic sequencing by oligonucleotide ligation and detection (SOLiD™, Applied Biosystems), and high-throughput DNA molecular cluster sequencing-by-synthesis using proprietary fluorescently labeled oligonucleotides (Illumina GAII Genome Analyzer, previously known as SOLEXA). These platforms have already demonstrated their significant worth: in the last two years, five new genomes have been read - in addition to the reference genome sequence that was determined by several leading research groups at institutes in the U.S., United Kingdom, and Canada in the course of 10 years and at a cost of 3 bln dollars [1] - such as the genomes of outstanding biologists of our times[2, 3], that of a Nigerian man [4, 5], a Chinese [6], and a Korean [7], not to mention thousands of other eukaryotic and prokaryotic species sequenced [8]. All these projects only became possible thanks to advanced technologies allowing cost-effective and high-throughput sequencing. We can expect an exponential increase in the amount of completely sequenced genomes in the near future; in particular, the recently launched 1,000 Genomes Project brings together multidisciplinary research teams from institutes around the world, including the United Kingdom, China, and the United States (http://www.1000genomes.org). Yet, despite considerable progress in the reading of DNA, the sequencing of large genomes, such as the human genome, has yet to become trivial. At present, there is no standardized approach to the analysis of these genomes, and how the performance of the above-mentioned technologies is assessed remains far from objective.

In the present work, we were the first in Russia to perform the full-genome sequencing of a Russian male using two advanced DNA sequencing technologies: Sequencing by Oligonucleotide Ligation and Detection (SOLiD™, Applied Biosystems) and DNA molecular-cluster sequencing with fluorescently labeled precursors (GAII, Illumina). In this work, we intended to optimize the algorithms of full-genome sequencing data acquisition, processing, and representation.

The general principles and terms governing large-scale advanced sequencing are as follows: initially, genomic DNA is subjected to fragmentation to obtain fairly short strands (200 to 1,000 basepairs (bp); then these fragments (*shotguns* - by analogy with the quasi-random firing pattern of a shotgun) are linked with oligonucleotide adapters, followed by PCR-amplification using specific enzymes and cloning for creating shotgun libraries. The library protocols for the GAII and SOLiD™ systems are described on the manufacturers' websites. Further procedures result in primary nucleotide sequences from each of the two ends of a DNA fragment from the library. These DNA sequences are called *reads*. The length of a read differs depending on the platform and is 36 nucleotides for GAII and 25 nucleotides for SOLiD™. Thus, each DNA fragment in the library is characterized by two reads with length and direction depending on the technological platform used. Then, the reads obtained by the sequencing of the shotgun library entries from the two ends (*paired-end reads*) are *mapped* to the human reference genome (hg18). This process, called *read mapping*, specifies the coordinate of the read in the genome. The mapping allows to draw the histograms of coverage and distance between paired-end reads and also to identify single-nucleotide polymorphisms (SNPs) and short insertions/deletions (indels). Moreover, the distance between the reads and their orientation might provide information on more considerable structural rearrangements in the studied genome. For instance, the essentially greater distance between the mapped reads compared to the overall length of the DNA fragments used for construction of the library is indicative of deletion between the reads in the analyzed genome, as compared to the reference one. Similarly, improper orientation of the reads that is discordant with the library design may suggest inversions in the analyzed region. Thus, large-scale genome sequencing using advanced technologies allows to determine short polymorphous loci and detect regions likely associated with great genomic abnormalities. However, the latter can be described only by *de novo* assembly of the reads into extended *contigs*, which was beyond this study.

## MATERIALS | METHODS

**DNA sample.** The male whose genome was to be sequenced was chosen from data contained in the Principal Component Analysis (PCA) of ethnic groups of the Russian Federation. One thousand three hundred eighty-two individuals representing 32 ethnic groups were genotyped by no less than 300,000 SNPs using high-density DNA microarrays. The group of ethnic Russians comprised 285 samples provided by Prof. D. G. Zaridze. The male whose genome was chosen for sequencing was characterized by principal components positioning him within the group of ethnic Russians on the 2D plot of the first vs. the second principal component (PC1-PC2). This PC1-PC2 area had no intersections with areas of other closely related ethnic groups (data in press).

**Sample preparation.** Genomic DNA was isolated from the arterial blood lymphocytes of a Russian male (patient N with renal cell carcinoma, see above, from the Blokhin Cancer Research Center, RAMS). The blood sample was collected with informed consent. The DNA was fragmented on a HydroShear® DNA shearing device (Genomic Solutions®, USA) to the average fragment size of 500 to 1,000 bp. Construction of genome libraries and all subsequent manipulations were carried out using supplemental reagent kits in accordance with the manufacturers' protocols. Both genome libraries were suitable for paired-end reading. Following adapter ligation, the genome library constructed for an Illumina Genome Analyzer II (GAII) DNA Sequencing Platform (Illumina, USA) was divided into two parts: the first was frozen, and the second one was used for PCR amplification (hereinafter Amplification No.1). When sequencing of this library was accomplished in nine flow cells, the second part was thawed and also subjected to PCR (Amplification No.2). These samples were sequenced in five flow cells.

The same fragmented DNA was also implicated in the construction of genome libraries suitable for paired-end reading on a SOLiD™ v.2 System (Applied Biosystems, USA) (hereinafter SOLiD). Following emulsion PCR in the reaction product (DNA immobilized on magnetic beads) was applied onto flow cells in which the ligase chain reaction was carried out. In each sequencing cycle, the enzyme (ligase) attached a fluorescently labeled oligonucleotide to the 5'-end of the substrate complex. Once identified, the fluorescence moiety was removed to produce the substrate complex elongated by five nucleotides. Over all, nine flow cells were used for sequencing.

**Sequencing.** Decoding of genetic information was performed using two platforms commercialized by Illumina, Inc., and Appied Biosystems, Inc. The first platform uses detection of fluorescently labeled nucleotides incorporated *in situ* into surface molecular clusters. This technology is embodied into an Illumina GAII DNA sequencing platform. The length of the reads is 36 nucleotides from each end, and 14 flow cells were used. The second technology, based on ligase chain reaction, is embodied into a SOLiD sequencing system. The length of the reads is 25 nucleotides from each end, and nine flow cells were used.

**Genotyping** of unfragmented genomic DNA was carried out using the Infinium technology on Illumina Human610-Quad BeadChips, according to the manufacturer's recommendations. The chips were scanned using an Illumina iScan System. Quality control has shown high conformity with the control parameters (call rate 99.7 %).Over all, allelic variants of 588,702 SNPs were reliably found. The SNP list is presented on the manufacturer's site (http://www.illumina.com/documents/products/marker_lists/marker_list_human660W_quad.zip).

**GAII data processing.** Analysis of the obtained images and their conversion to the DNA sequence was performed using the Illumina Genome Analyzer Pipeline v.1.4.0 software suite. The mapping of sequences to the human reference genome (hg18) was performed using the Eland program supplied with Genome Analyzer Pipeline and SOAPaligner/soap2 v.2.20 alignment program developed at the Beijing Genomics Institute (http://soap.genomics.org.cn/) (hereinafter SOAP, Short Oligonucleotide Analysis Package). The library of paired-end reads obtained from GAII is available at http://www.russiangenome.ru, the web site of the Project. This library allows to examine the localization and direction of the reads in genome browsers, such as the UCSC Genome browser or Ensembl Genome Browser available on demand. Nucleotide mismatches and short indels in the studied genome compared with the reference genome were calculated using the SOAPaligner/soap2.

**SOLiD data processing**. Pre-processing of SOLiD data for mapping was carried out using SOLiD software (provided as a component of the SOLiD System). Mapping of the sequences was carried out in initial color space using a SOLiD System Analysis Pipeline Tool (Corona Lite) v.4.0r2.0 and, following conversion from the color space to the FASTQ format, using a Burrows-Wheeler Aligner (BWA) v.0.5.1 installed on a computer cluster mounted at the RRC Kurchatov Institute. From 20 to 250 processing units were implicated in the data processing, depending on calculation complexity. Barring BWA, all calculations, both for GAII and SOLiD data, were carried out on dedicated computer of the above-mentioned computer cluster. Similarly to the GAII data, the data from SOLiD can be found on the site of the Project, http://www.russiangenome.ru, and viewed in genome browser.

**Original processing methods.** Calculations of coverage density, distances between the reads in paired-end libraries, and sequencing errors compared with those of genotyping on SNP microarrays were carried out using the authors' codes written on Perl. These codes are available on demand.

## RESULTS

**Processing of GAII and SOLiD datasets.**

**Fig. 1.**
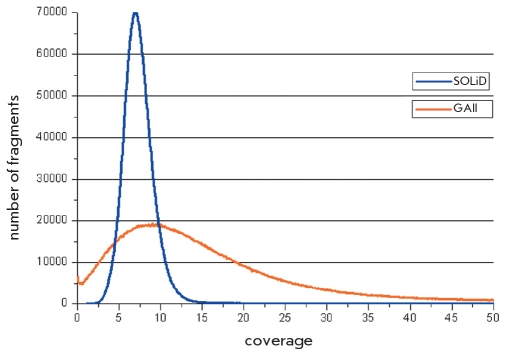
Genome coverage depth; GALL and SOLiD data are shown in orange and blue colors respectively

The total amount of data from Amplification No.1 and Amplification No.2 genome libraries, which passed the internal filters, was 60.2 Gbp or 1,674,748,960 reads for GAII and 48.1 Gbp or 1,926,071,502 reads for SOLiD. The mapping of reads to the human reference genome allowed to draw the coverage histogram (Fig.1). To do that, the genome was broken into tandem segments, 500 bp each, and the number of reads per segment was calculated from the mapping data (using Eland for GAII and BWA for SOLiD). This number was multiplied by the read's length (36 for GAII and 25 for SOLiD) and normalized by the fragment's length (500). The histogram of the GAII dataset has the shape of a Maxwell distribution with a peak at eightfold multiplicity and a tail shifted to tens of thousands of bp. This extremely high coverage largely corresponds to centromeric regions. Nucleotide-by-nucleotide analysis has shown that the GAII dataset covers, at least once, 2,033,881,571 nucleotides or 66.03% of the genome. General statistics of read mapping is presented in Table 1. It is worth noting that the amount of unmapped reads in the SOLiD dataset compared with the GAII one is almost twofold higher (32.65%).

**Table 1:** General statistic on the analysis performed. Percentages are in compliance with total read counts for either GAII (orange) or SOLiD (blue)

		GAII (SOAP)	SOLiD (CoronaLite)
Total nucleotides		60 290 962 560	48 151 787 550
Total reads		1 674 748 960	1 926 071 502
Total not mapped reads		17,41%	32,65%
Total single-end reads		13,99%	48,53%
Unique		5,75%	31,36%
Multiple		8,24%	17,17%
Mismatches	0	54,85%	55,67%
	1	16,95%	23,13%
	2	28,20%	21,20%
Total paired-end reads		68,60%	18,82%
Unique		51,16%	12,20%
Multiple		17,44%	6,62%
Mismatches	0	74,29%	28,14%
	1	16,32%	21,86%
	2	9,39%	50,00%
		**SOAP**	**CoronaLite**
Insertions (nucleotides)	Total	0,93%	-
	1	0,58%	-
	2	0,19%	-
	3	0,08%	-
	4	0,08%	-
Deletions (nucleotides)	Total	0,81%	-
	1	0,50%	-
	2.	0,17%	-
	3	0,07%	-
	4	0,07%	-

The score of nucleotide mismatches and short indels was only performed for unique alignments. Alignments were assumed as unique when mapped to the reference genome only once and, hence, characterized by unique location. About 82.6% of 1.67 billion reads corresponded (with minimal mismatch) to the reference genome sequences. The other 17.4% of reads were classified as unmapped. Random sampling inspection of 164 reads from this group has shown that none of them can be mapped to the reference genome with minimum distortion (less than two misalignments, indels no longer than four nucleotides). Over all, 13 out of 164 reads were attributed to other genomes: most to simian genomes, two reads to the genome of *Mus musculus*, and two reads to the genomes of *Danio rerio* and *E.coli*, respectively. However, all these fragments were not completely consistent with the DNA sequences of these organisms, whereas the major portion of unmapped reads showed complete, if rather short (no more than 25 nucleotides), coincidence with various human genome sequences.

**Checkup of matches between allelic SNP variants detected by sequencing and by genotyping on DNA microarrays.**

**Fig. 2.**
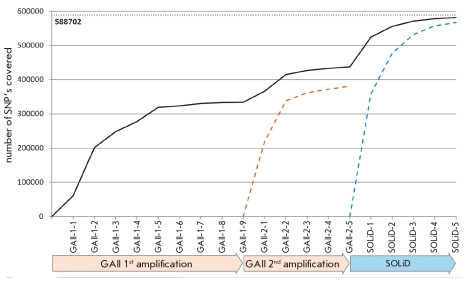
Coverage of genotyped SNPs by sequencing data and its saturation throughout the experiment. Shown is the number of SNPs at least once covered by reads mapped to the human reference genome ver. hg18 (by Eland for GAII and BWA for SOLiD data). Every step along the abscissa corresponds to the data from another sequencing run. Saturation only by GAII 2nd amplification runs is shown by orange dashed line. Saturation only by SOLiD runs is shown by blue dashed line

Using DNA microarrays, allelic variants of 588,705 SNPs with distinct locations were determined in the studied genome. For evaluation of the accuracy of SNP determination by the sequencing methodall reads encompassing the positions of microarray SNPs were selected (mSNP). The number of SNPs to which at least one read from the GAII and SOLiD datasets was mapped was 581,596 (98.8% of the total number of mSNPs). The reads from the GAII platform involved 437,056 SNPs (74% of mSMPs); those from SOLiD, 566,952 (96,3%). Sequencing of the genome libraries obtained at the stages Amplification No.1 and Amplification No.2 on the GAII platform resulted in reads overlapping 333,647 (56.7%) and 372,483 (55.6%) mSNPs, respectively (Fig.2). Homozygous mSNPs from Illumina 660W chip (totally 409,760) were only chosen to estimate the accuracy of allelic variant prediction by sequencing. Also, only mSNPs, to which no less than 1. 5 or 10 reads were mapped, were chosen. Their numbers are given in corresponding rows (coverage (≤ 1), coverage (≤ 5), and coverage (≤ 10)) in the Table 2. The same table mSNP percentages and the numbers of allelic variants that are closely similar - on different platforms and at different coverage levels - to the ones predicted by genotyping on DNA microarrays. We have found that sequencing allows to determine about 81% of mSNPs with an accuracy of no less than 95 % (Table 2, grey column "Eland or BWA," coverage ≤ 5).

**Table 2:** Comparison of homozygous SNPs detected by genotyping and inferred from sequencing data

		GAII (Eland)	SOLiD (BWA)	Both platforms (Eland or BWA)
Total homozygous SNP's on microchip		409760		
Tests performing	coverage (≥ 1)	302919	394373	404564
	coverage (≥ 5)	250353	238130	349309
	coverage (≥ 10)	194016	74902	270890
		After performing all tests		
Coverage ≥ 5	Amount	242201	218974	331873
	Percentage	96,74%	91,96%	95,01%
Coverage ≥ 10	Amount	188708	71999	261537
	Percentage	97,26%	96,12%	96,55%

**Analysis of information on paired-end reads.**

**Fig. 3.**
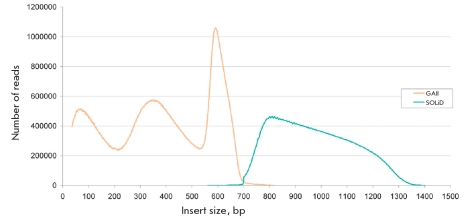
Paired-end reads' predicted insert size distribution; GAII and SOLiD data are shown in orange and in blue colors respectively. Reads were mapped to the human reference genome ver. hg18 (by Eland for GAII and Corona Lite for SOLiD data). The predicted insert size is a genomic distance between locations of mapped reads in each pair

The data on the relative position of paired-end reads on the reference genome was used for estimating the number of structural rearrangements in the studied genome. The plot of the number of paired-end reads mapped to hg18 versus the length of the reference sequence between the ends is shown on Figure 3. One can see on the figure that the lines significantly differ in shape between the two platforms, which can reflect the essential difference in the protocols used for construction of paired-end libraries. It is noteworthy that the GAII library distribution pattern of the distance between the paired ends shows local peaks at approximately 70 bp and 300 bp, as well as a high peak at 700 bp. These peaks are very likely associated with the reads mapped to DNA repeats of discrete lengths falling into these areas. Such a situation has already been described above as a problem encountered in the analysis of coverage histogram using ELAND. The plot of the distribution of the distances between reads in the SOLiD library shows a single peak at about 1,000 bp.

We also analyzed the possible three variants of the paired-end read's relative position and direction. The first variant is when the reads are mapped to the reference genome in accordance with the logic of library design. This means that for the GAII platform two reads face each other, if the 5'-3' direction is taken as the forward direction. As for the SOLiD platform, the logic of library design dictates unidirectional orientation of paired reads. The second and third variants are deviations from the "normal" position, which are only possible when essential rearrangements exist in the corresponding genome area. This leads to either one or two inversions in the paired-end read mapped to the reference genome. In accordance with this definition, all paired-end reads were subdivided into three classes: normal, carrying one inversion, and carrying two inversions (Table 3). Those reads that are mapped to different chromosomes of the reference genome were assigned to a separate class. A small excess of the reads mapped to different chromosomes is explained (in the case of SOLiD) by the presence of the blunt-end ligation stage that is implicitly fraught with errors associated with the covalent linkage of two fragments of different chromosomes. In general, both platforms are near-equal in the percentage of improperly oriented reads.

**Table 3:** Distribution of reciprocal paired end reads' position and orientation. A single inversion is considered in the case when one of the paired-end reads mapped to the human reference genome ver. hg 18 is reversed as against its espected orientation. A double reversion is the case when both reads are inverted. GAII and SOLiD data are shown in orange and blue colours respectively.

		GAII	SOLiD
Paired-end reads mapped to different chromosomes		3,18%	4,56%
Reciprocal position of paired reads:	Normal	96,12%	95,22%
	Single inversion	0,48%	0,14%
	Double inversion	0,22%	0,07%
Insertion sizes are in a permissible range		93,06%	95,43%

## DISCUSSION AND CONCLUSIONS

We have performed the full genome sequencing of an ethnic Russian male. The characteristic property of this work is the combination of two technological platforms: namely GAII and SOLiD. It is worth noting that data acquisition took approximately the same amount of time (eight weeks) on each platform. During that time, 14 launches (by one flow cell) of the GAII sequencer and 5 launches (by two flow cells) of the SOLiD sequencer were carried out. This allowed to identify 60.2 bln and 48.1 bln nucleotides, respectively. All working parameters of the technological platforms were within the nominal range (as stated by the manufacturers). The data were processed on a computer cluster of the RRC Kurchatov Institute, to which they were transmitted through a high-speed fiber optic cable. This required a special software module ensuring the integrity of the transmitted data. Primary data processing took about 10 weeks.

The main difference between the GAII and SOLiD datasets is in the evenness of the genome coverage by the reads produced by the sequencers. By our estimates, the GAII reads cover about 75% of the genome; and SOLiD reads, 95%, despite the fact that the GAII dataset was bigger compared with the SOLiD dataset. The same trend is observed when analyzing the histogram of genome coverage density (Fig.1). The peak value of the SOLiD dataset several times exceeds that of the GAII dataset. However, while the number of reads generated by SOLiD tends to zero already at a coverage density of 20 and more (blue line), the GAII dataset (orange line) has about 10,000 fragments in this range. Thus, the reads generated by the SOLiD system cover the reference genome essentially more uniformly than those generated by GAII. This reflects the quality of the shotgun library construction. The necessity to build several libraries, preferably differing in fragment lengths, for uniform coverage on the GAII platform was noted in studies performed by other research groups, particularly in the sequencing of the genome of a Korean [7]. Moreover, sequencing of two different PCR products of the same primary shotgun obtained at the preliminary step of library construction results in nonidentical, although largely superposed, coverage patterns. For instance, the curve of mSNP filling with reads achieved a plateau after nine launches of GAII with the Amplification No.1 library (Fig.2). The addition of the quasi-independent Amplificaton No.2 library resulted in a bounce of the plot, which reached another plateau after five launches of the sequencer. Thus, continuation of launches with the Amplification No.1 and Amplification No.2 libraries could not result in a consequent increase of genome coverage. The addition of the SOLiD dataset to the GAII dataset has solved the undercoverage problem. We suppose that this problem could also be solved on the GAII platform by building an additional library with another mean fragment size.

The sequencing accuracy was estimated by comparing the data on allelic variants of homozygous SNPs determined by sequencing with those determined by genotyping on a DNA microarray. When the SNP is covered by at least ten reads, the error in SNP determination is near equal in two platforms and is about 4-5%. However, the number of homozygous SNPs reliably identified by SOLiD compared with GAII is several times lower, because of insufficiently thick, although smooth, genome coverage by the reads.

The obtained data will allow to identify all SNPs of the given genome, SNP calling, and compare SNP allelic variants predicted by GAII and SOLiD. The analysis of the data on SNPs with fixed coordinates, whose allelic variants were determined by both sequencing and genotyping on a DNA microarray, shows that these results should largely have a high predictive potential and overlap no less than 95% of the time. Moreover, a comparison of all polymorphisms of the studied genome with those of already known genomes, particularly Craig Venter's and James Watson's genomes, is of undisputed interest. The second possible task would be to assemble *de novo* extended contigs from those reads that were not mapped to the reference genome. They may be indicative of DNA sequences that are not represented in the reference genome hg18, and further characterizing the genome we are studying.

Our data provide a background for further functional analysis of this genome. In particular, the data on heterozygous SNPs in expressed blood cell mRNAs will allow to determine transcripts with biallelic expression bias. This association between the epigenetic and genetic components of the given genome is of certain interest for further studies. However, this genome first has to become a wholesome model object, which can be achieved via immortalization of N's somatic cells followed by construction of the cell line. This will allow all interested research groups to use exhaustively the data presented in this work. 

## Acknowledgements

The study was supported by the Russian Federal Agency for Science and Innovation.
